# Towards Cervical Cancer Elimination: Insights from an In-Depth Regional Review of Patients with Cervical Cancer

**DOI:** 10.3390/curroncol33010052

**Published:** 2026-01-16

**Authors:** Anna N. Wilkinson, Kristin Wright, Colleen Savage, Dana Pearl, Elena Park, Wilma Hopman, Tara Baetz

**Affiliations:** 1 Department of Family Medicine, University of Ottawa, Ottawa, ON K1N 6N5, Canada; 2 Department of Oncology, Queen’s University, Kingston, ON K7L 2V7, Canada; kristin.wright@kingstonhsc.ca; 3 Research Security and Compliance Advisor, Queen’s University, Kingston, ON K7L 2V7, Canada; 4 Obstetrics and Gynecology, Queen’s University, Kingston, ON K7L 2V7, Canada; 5 Kingston Health Sciences Centre Research Institute, Kingston, ON K7L 2V7, Canada; wilma.hopman@kingstonhsc.ca; 6 Department of Public Health Sciences, Queen’s University, Kingston, ON K7L 2V7, Canada; 7 Department of Medicine, University of British Columbia, Vancouver, BC V6T 1Z4, Canada

**Keywords:** cervical cancer, cancer screening, HPV, pap test

## Abstract

Cervical cancer is a largely preventable disease, with over 90% of cases caused by persistent infection with human papillomavirus (HPV). Despite the availability of HPV vaccination and cervical screening, incidence rates in Canada have been rising since 2015, particularly among underserved populations. This study investigates contributing factors behind cervical cancer diagnoses in Eastern Ontario over a two-year period to identify gaps leading to failures in prevention and screening. A retrospective chart review was conducted for cervical cancer cases diagnosed between January 2022 and December 2023 at two regional cancer centres in Eastern Ontario. Cases were categorized as screen-detected, inadequately screened, or system failure, based on prior screening history and care processes. Data was collected on patient, screening, and cancer characteristics. Of 132 cases, 22 (16.7%) were screen-detected, 73 (55.3%) were inadequately screened, and 37 (28.0%) were attributed to healthcare system failure. Later-stage disease was significantly more common in the latter two groups. Thirty-one (23.5%) cases presented with palliative diagnoses, and 18 (13.6%) individuals died within 2.5 years. Inadequate screening was associated with rurality, deprivation, and lack of a primary care provider. System failures included false-negative Pap tests, loss to follow-up, and misapplication of screening guidelines. This study evaluated failures in cervical cancer prevention, which led to cervical cancer diagnoses in Eastern Ontario. Gaps included suboptimal screening participation, lack of access to care, health care system breakdowns, and limitations of the Pap test. Findings provide concrete suggestions for eliminating cervical cancer in Canada.

## Simple Summary

Cervical cancer is largely preventable, yet rates in Canada continue to rise. This study explores cervical cancer cases that were diagnosed in a two-year period in Eastern Ontario to better understand the factors leading to their development and identify opportunities to improve prevention. Cervical cancers were partitioned into three categories: cancers found through screening, cancers in people who were not adequately screened, and cancers linked to failures in the health care system. Of the 132 cervical cancers diagnosed during this two-year period, only about one in six were detected through screening. Nearly one quarter of the cases were incurable, and more than 13% of individuals died within two and a half years of diagnosis. Over half of the cases occurred in individuals who were inadequately screened. Lack of screening was more common among people living in rural areas, those experiencing social or economic disadvantage, and those without a regular primary care provider. System issues included false-negative Pap tests, loss to follow-up, and misapplication of screening guidelines. Overall, this study shows that gaps in access, follow-up, and screening quality continue to drive preventable cervical cancer in Canada and highlights clear opportunities to improve prevention and care.

## 1. Introduction

Cervical cancer is a largely preventable malignancy. More than 90% of cervical cancer cases are caused by the human papillomavirus (HPV) [1]. Persistent infection with oncogenic HPV strains can cause neoplastic transformation of cervical cells, which can progress from precancerous lesions to invasive cervical cancers. Prevention of cervical cancer relies on the synergy of primary prevention, with HPV vaccination, and secondary prevention, including cervical screening and prompt management of precancerous cervical changes. HPV vaccination has decreased the incidence of cervical cancer by 87% in immunized individuals, but this vaccination was only introduced in 2007 in Ontario [2,3]. Given that cervical cancer is a preventable malignancy, the Canadian Partnership Against Cancer aims to eliminate cervical cancer in Canada by 2040, and the World Health Organization has set a goal of eliminating cervical cancer globally [4].

Cervical cancer rates in Canada had been declining for over three decades. Unfortunately, since 2015, it is the fastest increasing cancer among Canadian females, growing by 3.7% per year, particularly in individuals aged 35 to 54 [5,6]. Approximately half of all cervical cancers occur in inadequately screened people [3]. The rise in cervical cancer incidence is higher in equity-deserving groups such as those living in rural or lower-income areas and Black women, who are more likely to be underscreened [2,3,7].

Canadian cervical screening guidelines, established in 2013, recommend screening with the Papanicolaou (Pap) test every three years and do not recommend HPV screening or HPV self-collection [8]. However, the sensitivity of the Pap test is low at only 55.4%, contributing to the development of up to 30% of invasive cases [9,10,11]. HPV cervical screening has been recognized to be a superior test, with a sensitivity of 94.6% and a decreased overall cost [9,10,12]. The majority of provinces in Canada continue to use the Pap test for cervical screening. HPV screening with self-collection has been adopted in two provinces in Canada, and HPV screening with clinician collection was introduced in Ontario in March 2025 [13].

This study is a regional review that examined all newly diagnosed cervical cancer cases in a two-year period in Eastern Ontario to identify the factors contributing to the development of this largely preventable disease. We conducted an in-depth review of the cases and the clinical and systemic issues surrounding their diagnoses to identify contributing care gaps. Our goal was to identify actionable insights that could inform improvements in cervical cancer screening to support ongoing efforts towards its elimination.

## 2. Methods

All cervical cancer cases registered or with a first encounter for which it was recorded as the Most Responsible Diagnosis at the Southeast Regional Cancer Programme at the Kingston Health Science Centre and The Ottawa Hospital Cancer Centre were investigated from 1 January 2022 to 31 December 2023. Cases were defined by the diagnostic codes C530 (malignant neoplasm of endocervix), C531 (malignant neoplasm of exocervix), C538 (overlapping malignant lesion of cervix uteri), and C539 (malignant neoplasm of uterus, unspecified). Transfers of care were excluded. We use the term “woman” to align with existing literature and guidelines while acknowledging that individuals of all gender identities with a cervix may require screening. This study was deemed a quality assurance/improvement project and granted exceptions from Research Ethics Board (REB) review by both Queen’s Health Sciences REB (Exemption # 6043745) and The Ottawa Health Sciences Network REB as per the Tricouncil Policy Statement 2, Article 2.5. Approval was given for data from both regions to be merged. This study followed the SQUIRE 2.0 reporting guideline for quality improvement in health care.

A chart review was performed to determine patient demographics, cervical screening history, and cervical cancer information. Pathological stage was used where available; otherwise, clinical stage was employed. Cases were noted as either stage IA or greater than stage IA. Deaths were recorded up to 30 June 2024. Postal codes were mapped to Census Subdivision to determine the Rurality Index, % Low Income Adults from 2016 Census data, and Ontario Marginalization Index (ON-Marg) information [14,15,16]. Cases were dichotomized into rural (RIO ≥ 1) or urban (RIO = 0), and ON-Marg first to third quintiles were categorized as “low,” and fourth to fifth quintiles were “high” for factors.

Cervical cancer cases were partitioned into three categories. Screen-detected cancers were defined as asymptomatic cancers detected through an abnormal screening Pap performed within 42 months of diagnosis. This time period aligns with Cancer Care Ontario’s definition of screening within the recommended interval of 36 months, with a 6-month grace period [17]. Inadequate screening was defined as individuals who were overdue for screening, with no Pap performed within 42 months of diagnosis. Healthcare system failure cases either had normal cervical screening within the 42 months prior to diagnosis (consistent with a false-negative Pap test), or patients were incorrectly identified as not requiring screening, or an abnormal screening Pap with no timely treatment or follow-up, or falsely reassuring follow-up.

Data were collected in an Excel file and imported into IBM SPSS (version 29.0 for Windows, Armonk, NY, USA, 2024) for statistical analysis. Data were initially described with frequencies and percentages for categorical data and means for continuous data. Differences between categories were assessed using Pearson chi-square tests or Fisher’s exact test if there were fewer than 5 cases in one or more cells for categorical data and one-way ANOVA for continuous data. A *p*-value of ≤0.05 was used to determine statistical significance, and no adjustment was made for multiple comparisons.

## 3. Results

Of the 132 cancers seen in Eastern Ontario regional cancer centres during the two-year time frame, 22 (16.7%) were screen-detected, 73 (55.3%) were in inadequately screened individuals, and 37 (28.0%) were in the system failure group (Table 1).

The age for individuals with screen-detected cancers was younger (mean age 44.4 years; *p* = 0.014) compared to inadequate screening (53.8 years) or system failure cases (47.9 years). Thirteen (9.8%) of all cases were seen in women aged 70 and older. BMI, smoking status, comorbidities, treatment of dysplasia, and preexisting psychiatric diagnoses were not significantly different between groups. High smoking rates were seen, with 54.5%, 53.4%, and 44.4% of cases in ever-smokers in screen-detected, inadequately screened, and system failure groups, respectively. Twenty-four (18.2%) individuals had previous dysplasia treatment. All patients with screen-detected cancers had a primary care provider (PCP), while 17 (23.3%) of inadequately screened individuals and 1 individual (2.7%; *p* = 0.001) with a system failure cancer did not. Inadequately screened cancers were found more often in individuals living in rural areas (39, 54.2%) compared to system failure (14, 37.8%) and screen-detected (6, 27.3%; *p* = 0.05) cases. There was no significant difference in partitioned groups by the ON-Marg factors of instability, dependency, or ethnic concentration. However, deprivation was higher in inadequately screened (25, 35.2%) than in screen-detected (5, 22.7%) and system failure (4, 11.4%; *p* = 0.020). Half of all inadequately screened individuals (49.3%) had no Pap within 10 years of diagnosis. Three cases were diagnosed in individuals who had immigrated within five years of diagnosis who had either never been screened or had been lost to follow-up. Normal Pap results in the recommended interval prior to formal diagnosis with cervical cancer were seen in 25 (18.9%) of the 132 individuals.

None of the screen-detected cancers were diagnosed through the emergency department, compared to 28 (21.2%) of inadequately screened and system failure cases. Adenocarcinomas were most common in the screen-detected cases (8, 36.4%) compared to 17 (23.3%) in inadequately screened and 8 (21.6%) in system failure (*p* = 0.573); the ratio of squamous cell cancers to adenocarcinomas was highest in the system failure group. Cases that were greater than IA were noted in 60.6% of cases and were significantly more common in inadequately screened (47, 64.4%) and system failure (30, 81.1%) than screen-detected (3, 13.6%; *p* < 0.01). Proportions of palliative, or stage IV, diagnoses were significantly different between groups (*p* = 0.030), with no such diagnoses occurring in the screen-detected group and 18 (24.7%) and 13 (35.1%) in the inadequately screened and system failure groups, respectively. Eighteen individuals (13.6%) died by 30 June 2024.

Reasons for inadequate screening determined through chart review included reluctance to engage in screening due to a history of sexual abuse, substance abuse, pelvic pain, or agoraphobia. Barriers to screening participation, such as no PCP and cessation of screening activities due to the COVID-19 pandemic, were also noted. The etiology of system failure cases included false-negative Pap tests or colposcopy, individuals who were lost to follow-up or lost results, and inadequate identification of eligibility. Individuals not recognized as eligible for screening included those with incomplete fulfillment of criteria for screening cessation at age 69 (i.e., 3 normal Pap tests within the previous 10 years), misunderstanding around the presence of a cervix after subtotal hysterectomy, and transmasculine individuals with cervix in situ.

**Figure 1:**
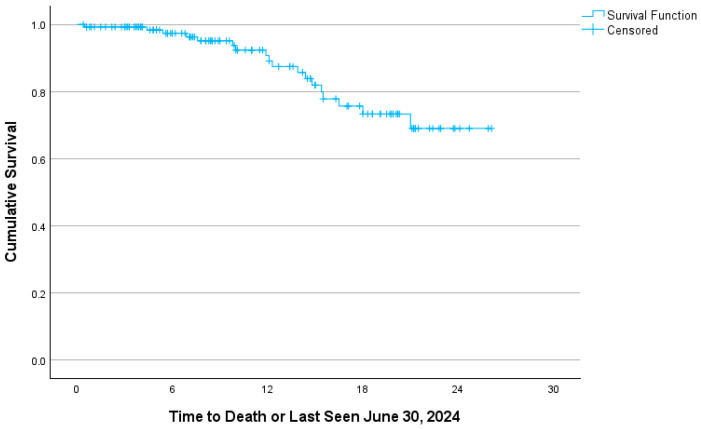
Cervical cancer survival. Kaplan–Meier survival curves for Eastern Ontario cervical cancer cases diagnosed from 1 January 2022 to 31 December 2023 and followed until 30 June 2024.

**Figure 2:**
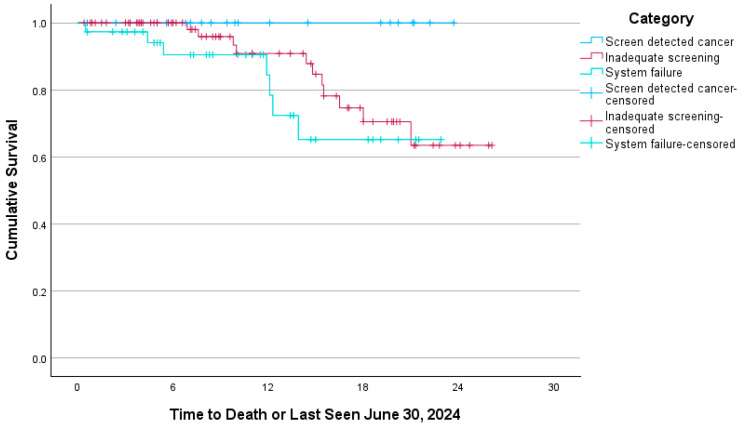
Cervical cancer survival according to detection category. Kaplan–Meier survival curves by screen-detected, inadequate screening, and healthcare system failure categories. Screen-detected: Asymptomatic cancers detected through an abnormal screening Pap performed within 42 months of diagnosis. Inadequate screening: Individuals who were overdue for screening, with no Pap performed within 42 months of diagnosis. Healthcare system failure: Normal cervical screening within the 42 months prior to diagnosis, or lack of identification of the need for screening, or abnormal screening Pap with no timely treatment or follow-up.

Overall survival was 91.7% at two years and 86.4% after an additional six months of follow-up (Figure 1). Survival curves between categories approached significance (*p* = 0.082), with screen-detected cancer demonstrating better survival (Figure 2). Median survival time was not reached.

## 4. Interpretation

This study provides critical insights into the patient and system issues that led to the failure of cervical screening in Eastern Ontario. There were 132 cases of almost entirely preventable invasive cervical cancer cases with a 13.6% mortality rate within 2.5 years. Even the screen-detected cancers noted in this study, albeit being less advanced, represent a failure of screening, as these should have been avoided through treatment at the preinvasive stage. Cancers that were not screen-detected due to inadequate screening or system failure were more frequently later stage, with up to a third diagnosed at stage IV. Characteristics of inadequately screened individuals included rural location, lower socioeconomic status, and a quarter of these individuals did not have a PCP. Patient factors, such as a history of sexual abuse, pelvic pain, or anxiety, contributed to reluctance to engage in screening. Just over a quarter of cases were due to preventable system issues such as lost to follow-up, missing results, false-negative Pap tests, and inappropriate cessation of screening. These results highlight significant gaps in cervical screening at a programme level, which align with those noted in the “White Report” (Box 1) [18].

Box 1 **Recommendations to improve cervical screening based on identified care gaps.**

• Increase HPV vaccination.
• Encourage smoking cessation.
• Adopt the higher sensitivity HPV screening test.
• Adopt HPV self-testing to address patient barriers to engagement, geography, and access to primary care.
• Offer opportunities for cervical screening for unattached individuals.
• Ensure access to cervical screening for new immigrants and refugees.
• Consider equity-focused screening delivery to ensure uptake by populations underserved by current practices, i.e., psychiatric diagnoses, rural, and low socio-economic status.
• Strengthen cervical screening registries.
• Employ patient-specific electronic reminders to prompt engagement and decrease loss to follow-up.
• Ensure transgender individuals receive appropriate screening.
• Ensure ongoing screening of patients treated for cervical dysplasia who remain at elevated risk.
• Ensure appropriate cessation of screening.
• Consider extending screening beyond age 69, depending on the individual’s overall health and functional status, clinical history, and risk factors.
• Implement opportunistic screening at medical touchpoints involving the cervix, including therapeutic abortions, IUD insertions, and pregnancy care.

This study highlights important limitations of the Pap test, which are cause for concern. Nineteen percent of women studied with cervical cancer had normal, up-to-date screening results, consistent with the previously noted 15–65% false-negative Pap test rate in the context of screening programmes [19]. As well, although adenocarcinomas typically represent only 10% of cervical cancer cases, in this study, over a third of cases were adenocarcinomas, likely because they are not optimally diagnosed with Pap tests, given their endocervical nature [20]. Our high false-negative rate and evidence of poor diagnosis of adenocarcinomas with the Pap test emphasize the necessity of adopting HPV testing, given that it has been proven to provide superior protection against cervical cancer compared to cytology [21]. A continued reliance on cytology for cervical screening, with its low sensitivity and longer intervals to detection, will result in the ongoing development of potentially preventable cervical cancers, as was observed in this regional review. Canada’s slow and uncoordinated uptake of HPV screening and self-collection due to out-of-date national guidelines and the resultant variation in provincial implementation may contribute substantially to our observed healthcare system failure in cervical cancer cases.

The high false-negative cytology and lapses in follow-up, as well as later-stage cancers at diagnosis, noted in our study, have been previously noted in the literature [22]. A review of cervical cancers in a Danish population-based cervical screening programme found that 45.5% of women had inadequate screening, while their rate of false-negative Pap tests was higher at 40.2% of individuals [23]. A Canadian study from 2007 found that only 16% of individuals had proof of regular screening, suggesting that failure to recruit to screening was the largest contributor to the development of cervical cancer [24].

Inadequate screening accounted for a large proportion of the cervical cancer cases. Previous studies have found that 30–60% of cervical cancer cases are found in people with inadequate screening [3,19,24]. PCP attachment rates were significantly lower among individuals with inadequate screening, highlighting the impact of the current primary care crisis on access to screening and its trickle-down impact on later-stage cervical cancer and higher mortality. HPV self-sampling presents a means to mitigate the lack of access to primary care if distributed through a centralized system [25]. Self-sampling would also address additional patient-level barriers to screening noted in this study, such as rurality, a history of sexual abuse, pelvic pain, and anxiety, as well as the known suboptimal screening in women with a greater BMI due to sampling error [26]. Opportunistic screening could be considered to improve screening access, as we noted women who were parous had undergone therapeutic abortions or IUD insertions in a timeframe close to diagnosis. These individuals had clearly had interactions with the health system involving their cervix prior to diagnosis, reflecting missed opportunities to identify and treat dysplasia.

We noted no increase in cervical cancer cases in ON-Marg quintiles with high racial and ethnic concentrations in our study. However, we did observe cervical cancer in a few newly immigrated women who did not have access to screening in their country of origin, aligning with other studies, which found that immigrant women are less likely to be screened [27,28]. The lack of cervical screening and/or inadequate follow-up seen in cervical cancer cases in new immigrants in this study underlines the importance of ensuring access to and education around cervical screening through newcomer clinics and subsequent attachment of these individuals to primary care. Cervical cancer rates in the US are higher in populations with lower education, higher rurality, and lower socioeconomic status [3,19]. The cohorts in this study who were overdue for screening or experienced system failure displayed similar trends with significantly higher maternal deprivation and rurality.

This study highlights several system issues with cervical screening. Eighteen percent of cases occurred in women previously treated for dysplasia. Cervical cancer risk post-dysplasia treatment continues to climb over time, with an HR of 2.56 in these individuals 25 years post-treatment [29]. US guidelines suggest that women treated for dysplasia should be screened for a minimum of 25 years after treatment [30]. However, documented HPV-based test-of-cure has been shown to reduce the increased risk of invasive cervical cancer after treatment for dysplasia [31,32]. The rates of cervical cancer after dysplasia treatment in our study did not reflect the benefit of HPV testing and provided further impetus for the transition to HPV screening.

In a recent study from California, one in five cervical cancer cases was seen in women older than 65, and these older women had significantly later stage at diagnosis and worse survival than younger women [33]. Considering that almost 10% of our cases were found in women older than 70, clearer documentation and education around screening cessation parameters are necessary to ensure that women have had the required negative screens to cease screening. As well, given longer lifespans and ongoing sexual activity in older age, consideration should be given to the role of screening in individuals who have had new exposure to HPV after screening cessation (which, with HPV screening, could be after age 65). In the context of a life expectancy longer than 10 years, there could potentially be time for cervical cancer to develop. Additionally, cytology interpretation can be more challenging in the context of atrophic changes [34]. As such, even with a transition to HPV screening, the use of cytology triage in this population should be carefully assessed, and the use of objective markers such as HPV genotyping preferentially employed.

Increased training and education for providers in regard to cervical screening guidelines could improve cervical cancer outcomes. System failure was found to exist due to knowledge gaps around screening in those with a previous subtotal hysterectomy or transgender individuals with a cervix remaining in situ. These cases reflect gaps in both clinician awareness and systemic tracking of cervical status and screening eligibility. Increased knowledge of appropriate candidates for screening, especially with respect to transgender individuals, could improve uptake of screening. A clearer understanding of the recommended follow-up for those with abnormal screens, including adequate training for colposcopy, is important, as well as a clear understanding of the necessary parameters for screening cessation. In addition, the loss to follow-up cases and/or results indicates a need for strengthened screening registries and modernized electronic screening or follow-up reminders.

Limitations of our study include its retrospective chart review design, which may have resulted in incomplete data capture. Certain variables, such as psychiatric history, comorbidities, and smoking status, may not have been consistently documented. Additionally, HPV vaccination status could not be reliably determined, although some individuals in this study had been vaccinated, highlighting the ongoing need for cervical screening in this population. It is also important to note that cervical cancer screening programmes were paused during the COVID-19 pandemic, which may have contributed to the number of overdue cervical screening tests observed in our cohort. System failures occurred in almost a third of patients, including false-negative results, loss to follow-up, or inadequate follow-up. The latter two may have been due to patient factors, provider factors, system factors, or a combination. Due to the retrospective chart review design, we were not able to reliably differentiate between these. Future directions for research would include a similar retrospective chart review in the setting of ongoing HPV screening to assess for improved outcomes.

## 5. Conclusions

This study is a call to action—132 women in Eastern Ontario were diagnosed with cervical cancer, and there were 18 deaths from this preventable disease in a short timeframe. The associated morbidity, mortality, and implications for system resource use for these cases are disturbing. The gaps identified in this study provide a roadmap to improve cervical screening. HPV vaccination and smoking cessation must be strongly encouraged. We must move nationally to implement the more sensitive HPV screening test and HPV self-collection. There must be targeted outreach to underserved populations and improved access for unattached patients, including opportunistic screening, especially for inadequately screened individuals. Strengthened registry infrastructure could ensure timely follow-up, appropriate surveillance after dysplasia treatment, and accurate application of cessation criteria. These recommendations range from simple to system-wide, yet they have enormous potential to help move the needle closer to the eradication of cervical cancer.

## Figures and Tables

**Table 1:** Patient, screening, and cancer characteristics for cervical cancer cases by category.

	Category			
	Screen detected	Inadequate Screening	System failure	
	*n* = 22 (%)	*n* = 73 (%)	*n* = 37 (%)	
	**Mean**			***p*** **-value**
Age	44.4	53.8	47.9	**0.014**
BMI	22.0	25.2	24.5	0.151
	***N***			
BMI Underweight (<18.5)	5 (22.7)	10 (13.7)	10 (27.0)	0.196
BMI Healthy weight (18.5–24.9)	12 (54.5)	29 (39.7)	14 (37.8)	
BMI Overweight (25.0–29.9)	3 (13.6)	20 (27.4)	4 (10.8)	
BMI Obese (30.0+)	2 (9.1)	14 (19.2)	8 (21.6)	
Cases diagnosed after age 70	0 (0.0)	10 (13.7)	3 (8.1)	0.170
Ever smoker	12 (54.5)	39 (53.4)	16 (43.2)	0.637
At least one comorbidity	8 (36.4)	36 (49.3)	13 (35.1)	0.223
Pre-existing psychiatric diagnosis	6 (27.3)	23 (31.5)	8 (21.6)	0.550
No primary care provider	0 (0.0)	17 (23.3)	1 (2.7)	**0.001**
History of dysplasia treatment	6 (27.3)	12 (16.4)	6 (16.2)	0.480
Rural	6 (27.3)	39 (53.4)	14 (37.8)	**0.050**
ON-Marg High instability	8 (36.4)	31 (42.5)	10 (27.0)	0.316
ON-Marg High deprivation	5 (22.7)	25 (34.2)	4 (10.8)	**0.026**
ON-Marg High dependency	9 (40.9)	43 (58.9)	18 (48.6)	0.244
ON-Marg High ethnic concentration	4 (18.2)	7 (9.6)	2 (5.4)	0.340
No pap within 10 years of dx	0 (0.0)	36 (49.3)	7 (18.9)	**<0.001**
No pap with 42 months of dx	1 (4.5)	68 (93.3)	14 (37.8)	**<0.001**
Symptomatic presentation	4 (18.2)	52 (71.2)	27 (73.0)	**<0.001**
Diagnosed through the ED	0 (0.0)	21 (28.8)	7 (18.9)	**0.006**
				
Stage > 1A	3 (13.6)	47 (64.4)	30 (81.1)	**<0.001**
Histology: Adenocarcinoma	8 (36.4)	17 (23.3)	8 (21.6)	0.573
Squamous cell	12 (54.5)	52 (71.2)	26 (70.3)	
Other	2 (9.1)	4 (5.5)	3 (8.1)	
SCC: Adenocarcinoma ratio	1.5	3.1	3.3	NA
Palliative diagnosis	0 (0.0)	18 (24.7)	13 (35.1)	**0.003**
Died by 30 June 2024	0 (0.0)	11 (15.1)	7 (18.9)	0.075

SD = standard deviation; BMI = body mass index; ON-Marg: Ontario Marginalization Index; ED = emergency department.

## Data Availability

The original contributions presented in this study are included in the article. Further inquiries can be directed to the corresponding author.
